# A Rarely Reported Case of Enterococcus faecalis Bacteremia Causing Infective Endocarditis and Osteomyelitis

**DOI:** 10.7759/cureus.22522

**Published:** 2022-02-23

**Authors:** Pradeep Kumar Devarakonda, Vishal R Dhulipala, Monika Karki, Cesar Ayala-Rodriguez, Sarath Reddy

**Affiliations:** 1 Internal Medicine, The Brooklyn Hospital Center, Brooklyn, USA; 2 Internal Medicine, Brooklyn Hospital, Brooklyn, USA; 3 Cardiology, The Mount Sinai Hospital, New York, USA; 4 Cardiology, Brooklyn Hospital Center-Mount Sinai Heart, Brooklyn, USA

**Keywords:** bioprosthetic valve, muskuloskeletal, enterococcus faecalis, endocarditis, infective endocarditis, aortic endocarditis, osteomyelitis, osteomyelitis treatment, rare, rare association

## Abstract

Infective endocarditis (IE) is an infection of the heart valves or endocardium, usually due to the spread of infection through the blood. It can cause a varied range of symptoms, from being asymptomatic to reduced heart function, valvular abnormalities, embolization, or death. Enterococci are usually present as normal gut flora but can also cause bacteremia, urinary tract infections, or IE, especially in the elderly population. The source of enterococcal spread in most of the cases is unidentifiable and sometimes associated with the genitourinary tract or damage to the gut mucosa due to trauma, malignancy, and infection, among others. Very few cases have been reported so far on *Enterococcus faecalis *(*E.faecalis*) endocarditis and even rarer for such cases complicated by osteomyelitis. Here, we describe the case of a 63-year-old male patient with a recent history of cardiac arrest, a percutaneous endoscopic gastrostomy tube placement, and endotracheal tube placement. He presented with back pain and was found to have osteomyelitis on magnetic resonance imaging and aortic valve vegetations on transthoracic echocardiography (TTE). His blood cultures were positive for *E.faecalis*. Repeat TTE showed growth in the vegetation, and the patient underwent bioprosthetic aortic valve replacement.

## Introduction

Infective endocarditis (IE) is one of the less frequent infections, with an annual incidence of five to seven cases per 1,000,000 people. *Enterococcus* is a genus of gram-positive, facultatively anaerobic bacteria that were previously classified as group D *Streptococcus*, currently a separate genus. *Enterococcus faecalis *and *Enterococcus faecium*, which ordinarily inhabit the gastrointestinal, hepatobiliary, and genitourinary flora, are the most prevalent pathogenic subspecies. *E. faecalis* is the third most prevalent cause of IE, accounting for around 5% to 15% of all cases [1,2]. However, enterococcal infections with complications of osteomyelitis are rare. We thus describe one such case in this report.

## Case presentation

**Figure 1:**
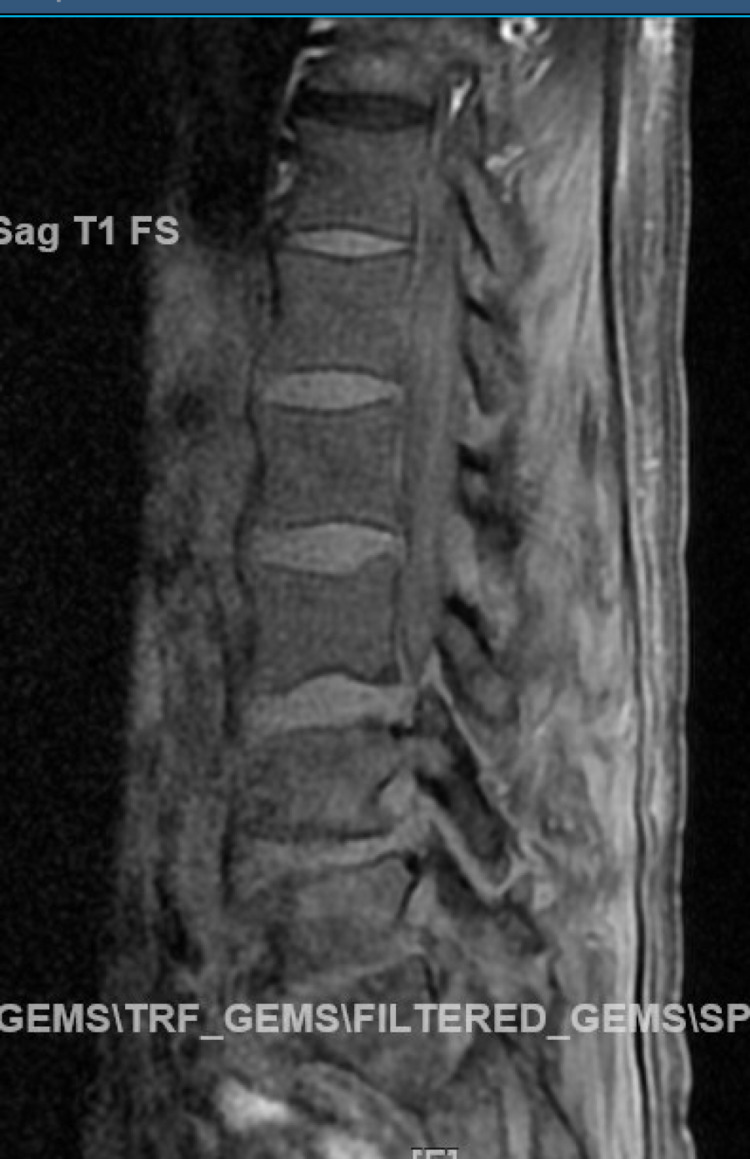
Mild enhancement is seen in the vertebral endplates at L5-S1 in the region of endplate edema.

**Figure 2:**
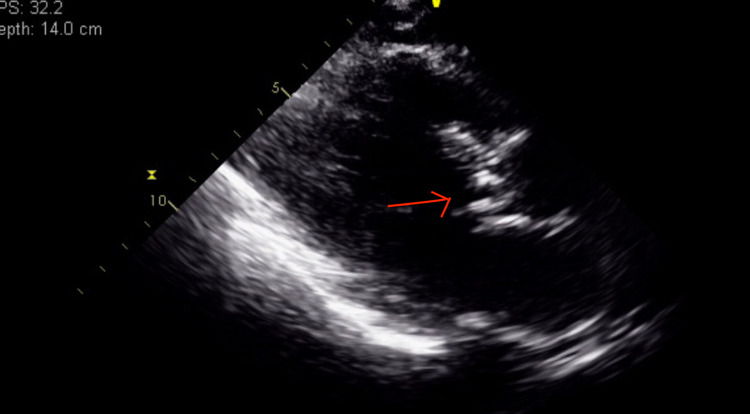
PLAX-vegetations on the right and non-coronary cusps of aortic leaflets on the ventricular side at the start of systole. PLAX: parasternal long-axis view.

**Figure 3:**
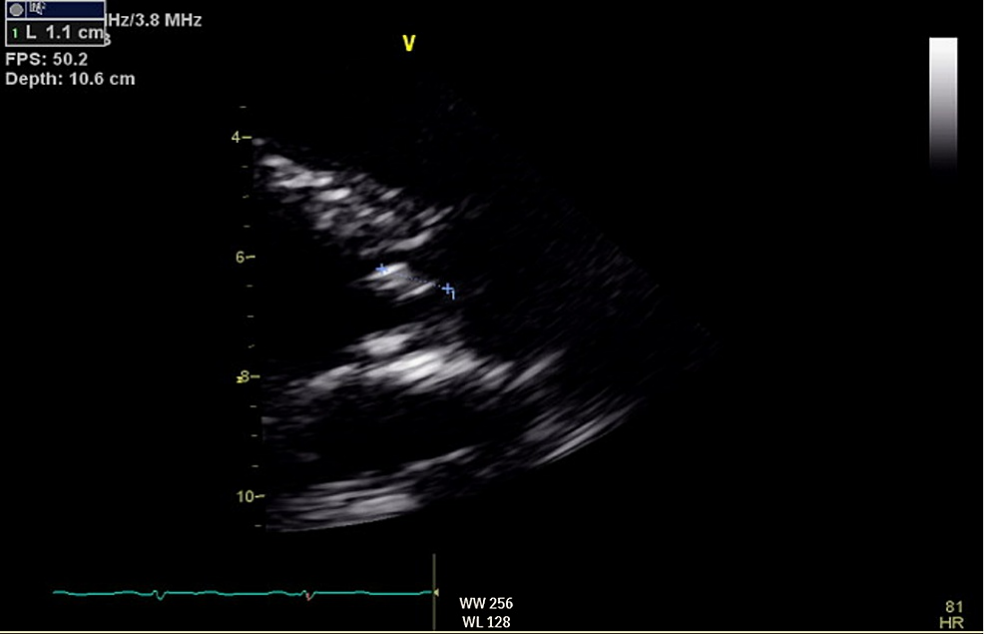
PLAX view-vegetation measuring 1.1 cm on the right coronary cusp. PLAX: parasternal long-axis view.

**Figure 4:**
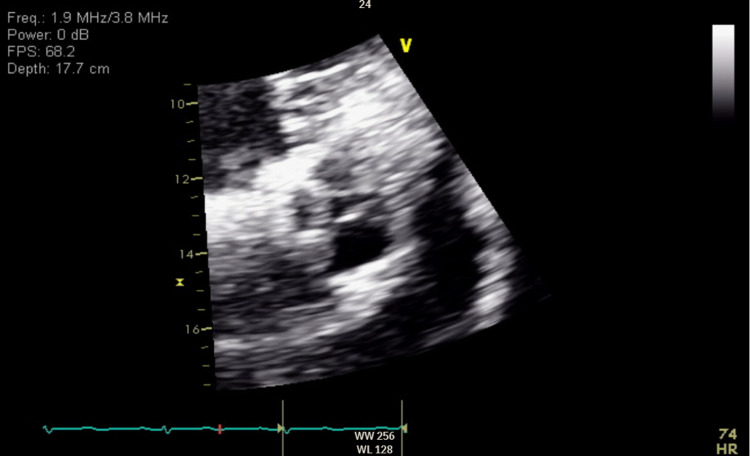
PSAX view zoomed on the aortic valve. PSAX: parasternal short axis.

**Figure 5:**
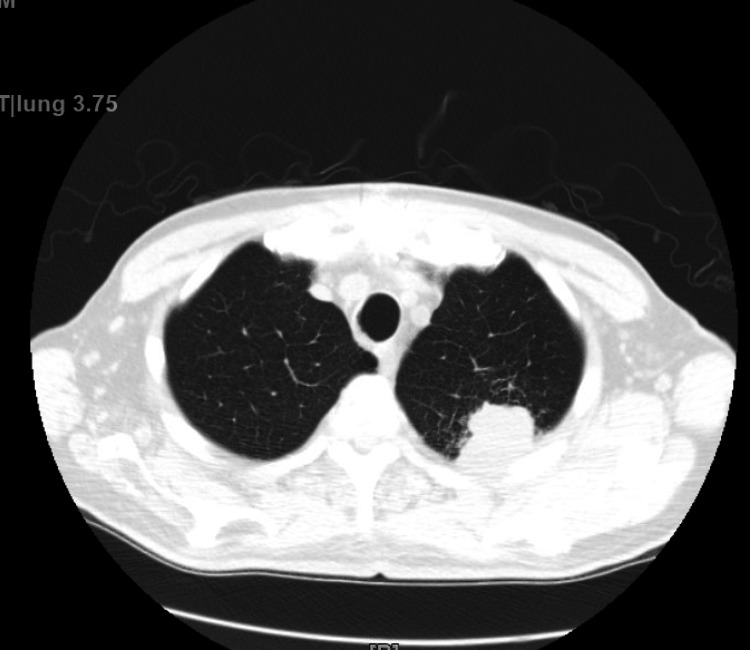
There is a mass in the posterior aspect of the left apex that is inseparable from the pleura. This mass measures approximately 4.4 × 4.3 × 2.8 cm.

A 63-year-old male patient with a previous medical history of hypertension, non-obstructive coronary artery disease, cocaine abuse, and a history of cardiac arrest two months back, presented with intractable lower back pain for four days. The patient's previous hospitalization was complicated by bilateral pneumothoraces, chest tube, and percutaneous endoscopic gastrostomy tube placements, followed by discharge after removal of all tubes. This time, the patient had no other symptoms other than back pain, such as motor or sensory loss, urinating or defecating difficulties, or saddle anesthesia, but was unable to walk and suffered from severe back pain. Lumbar magnetic resonance imaging showed soft tissue swelling at L5-S1, indicative of osteomyelitis, Figure 1. The patient was started on vancomycin initially and later changed to ampicillin and ceftriaxone after blood culture results became positive for *E. faecalis*. Transthoracic echocardiography (TTE) was performed considering the risk factors, and it showed normal left ventricular ejection fraction (LVEF), a mildly dilated left atrium, moderate aortic regurgitation, moderate-sized vegetation on the aortic valve, and mild to moderate tricuspid regurgitation. Repeat focused TTE showed two 1-cm hypermobile echo densities on the ventricular side of the right aortic valve and a non-coronary cusp, likely vegetation, Figures 2-4. Chest computed tomography also showed a mass on the left upper lobe, inseparable from the posterior pleura and 4.4 × 4.3 × 2.8 cm, Figure 5. However, the patient refused all the workup, including a biopsy, to rule out septic emboli or malignancy. The patient also had one episode of bloody bowel movement, with the hemoglobin level at 6.9 (A1) gm/dl. However, the patient refused endoscopy and colonoscopy. His hemoglobin subsequently improved without any events. The patient continued on ceftriaxone and ampicillin and eventually underwent a bioprosthetic aortic valve replacement.

## Discussion

IE was first described by William Osler in 1885. It is one of the less frequent infections affecting the heart valves or endocardium, with high mortality and morbidity. *E. faecalis* is a type of catalase-negative, gram-positive, facultative anaerobe, often appearing as diplococci in short chains under microscopy [1]. *E. faecalis* IE usually has a high incidence in the aging population, likely due to increased procedures involving the gastrointestinal and urinary tracts, heart valve pathologies, or implanted prosthetic material for the heart [3]. *E. faecalis* IE has become a significant infection, considering the increasing frequency of hospital-associated infections and increasing resistance to antibacterial medications [4].

In 2003, Nicholas at the Mayo Clinic showed how rare *Enterococcus*-associated osteomyelitis is. Among 462 patients who were treated at the Mayo Clinic from 1969 to 1991 for infected prosthetic joints without IE, only six cases (1.3%) were *Enterococcus*-associated infections. IE cases treated at the Mayo Clinic from 1965 to 1975 totaled 192 patients, out of which 84 (44%) had musculoskeletal complaints, 5 (6%) had possible osteomyelitis, and only one had *Enterococcus* as a causative organism [5]. In a retrospective study conducted on IE by Sapico et al., 24 episodes (23%) of musculoskeletal complaints were observed. Of the 104 IE cases examined, 15 (15%) had an osteoarticular infection, which suggests an increased risk for osteomyelitis among infected endocarditis patients [6].

The diagnosis of IE requires clinical, laboratory, and imaging examinations. The patient in the present case was diagnosed with IE as two major Duke criteria were positive, i.e., this patient had an episode of bloody bowel movement, but the patient did not have endoscopy or colonoscopy. So colorectal carcinoma was one of the differentials that needed to be ruled out. If the patient had aortic stenosis, Heyde syndrome should be considered in differentials since a triad of aortic stenosis, angiodysplasia, and anemia makes Hyde syndrome more likely. A case of *E. faecalis* endocarditis in a patient with Heyde syndrome has been reported [7]. This patient did not have any aortic stenosis, and angiodysplasia could not be diagnosed. Even though musculoskeletal complaints are common in IE patients with localized complaints, clinicians should also suspect osteomyelitis. 

A combination regimen of aminoglycosides like gentamycin with ampicillin was initially considered and was the standard treatment for *E. faecalis* endocarditis for several years. However, due to the rise of antimicrobial resistance and nephrotoxicity, an alternate therapy of ampicillin and ceftriaxone for four to six weeks is recommended. In 1995, Mainardi et al. initially reported the synergistic effect of ampicillin and ceftriaxone, which was later confirmed by Gavaldà et al. and Fernández-Hidalgo as an equally effective and safe alternative, especially in patients with renal failure [8,9].

## Conclusions

IE is a rare and life-threatening cardiac infection with high mortality and morbidity. *Enterococcus* is the third leading cause of IE and can present with musculoskeletal complaints. Even though these complaints are common, sufficient evaluations need to be performed to rule out osteomyelitis. Colorectal carcinoma should also be ruled out if the patient presents risk factors or suspicion. Early diagnosis and treatment with dual beta-lactams improve survival.
